# Insanity in Woman

**Published:** 1871-07

**Authors:** 


### "INSANITY IN WOMEN."

"BY H. R. STORER, M. D."

This valuable work presents itself in such a concise form, that even in this day of multiplicity of good books, one is well paid for its careful perusal. A treatise on this subject, giving the experience and convictions of so eminent a person as the author, is truly welcome.

The object of the work is to prove that a large proportion of cases of insanity in women is of a reflex nature, arising from diseases of the pelvic organs; and having proved this, to indicate the only rational mode of treatment.

He corroborates his views by giving the testimony of prominent psychological experts, so that much exceedingly valuable information is given.

His proposed treatment is not the one usually followed, and we would call particular attention to the fact, that on pages two hundred and thirteen and fourteen, there is an overwhelming argument in favor of having a *woman* physician placed in charge of the female wards in all insane asylums.

A thought, that made a deep impression during the perusal of this work, is this: If disease of the pelvic organs in woman, causes even insanity, why could not some influences be set at work, that would result in women so living and dressing that those parts could be maintained in a state of health. That this can be done, with woman's present skirts and waists, is utterly impossible. It is safe to assert, that no one who has worn them, can have all the internal parts in a perfectly normal state. Soft organs will succumb to constant pressure, even though it is but slight.

We object entirely to the idea that woman is even delicate on account of her sex. She is naturally very strong, or how could the race be continued.

One point of interest in this work, is that it gives evidence in favor of the natural powers of endurance possessed by women. Her greater tenacity of life shows itself even when disease has such a firm hold of her body as to dethrone reason.

He says, "the relative mortality of insane women is less than of insane men." He also states that Legoyt attributes this difference to "the greater vitality" of the female sex.

Out of this "greater vitality" surely something better ought to be developed, than the weak backed women of to-day.

That person who, by some maneuver, shall make fashionable a costume for woman, that would leave her body as free from weight and pressure, as a man's body is in his costume, would confer a greater boon on the race than the discovery of chloroform. It would save half the race from backache, nip a host of evils in the bud, and succeeding generations would have cause for infinite gratitude.

In the meantime, however, while the constitution of woman is being steadily undermined, thanks are due to Dr. Storer for calling attention to the most alarming of all results of disease of the pelvic organs, and for doing all in his power to alleviate distress. C.

